# The *atm*-*1* gene is required for genome stability in *Caenorhabditis elegans*

**DOI:** 10.1007/s00438-012-0681-0

**Published:** 2012-02-18

**Authors:** Martin R. Jones, Jim Chin Huang, Shu Yi Chua, David L. Baillie, Ann M. Rose

**Affiliations:** 1 Department of Medical Genetics, University of British Columbia, 419-2125 East Mall, Vancouver, BC V6T 1Z4 Canada; 2 Molecular Biology and Biochemistry, Simon Fraser University, Burnaby, BC V5A 1S6 Canada

**Keywords:** DNA repair, Mutator, ATM, *C. elegans*

## Abstract

The Ataxia-telangiectasia-mutated (ATM) gene in humans was identified as the basis of a rare autosomal disorder leading to cancer susceptibility and is now well known as an important signal transducer in response to DNA damage. An approach to understanding the conserved functions of this gene is provided by the model system, *Caenorhabditis elegans*. In this paper we describe the structure and loss of function phenotype of the ortholog *atm*-*1*. Using bioinformatic and molecular analysis we show that the *atm*-*1* gene was previously misannotated. We find that the transcript is in fact a product of three gene predictions, Y48G1BL.2 (*atm*-*1*), K10E9.1, and F56C11.4 that together make up the complete coding region of ATM-1. We also characterize animals that are mutant for two available knockout alleles, *gk186* and *tm5027*. As expected, *atm*-*1* mutant animals are sensitive to ionizing radiation. In addition, however, *atm*-*1* mutants also display phenotypes associated with genomic instability, including low brood size, reduced viability and sterility. We document several chromosomal fusions arising from *atm*-*1* mutant animals. This is the first time a mutator phenotype has been described for *atm*-*1* in *C. elegans*. Finally we demonstrate the use of a balancer system to screen for and capture *atm*-*1-*derived mutational events. Our study establishes *C. elegans* as a model for the study of ATM as a mutator potentially leading to the development of screens to identify therapeutic targets in humans.

**Electronic supplementary material:**

The online version of this article (doi:10.1007/s00438-012-0681-0) contains supplementary material, which is available to authorized users.

## Introduction

A wide range of proteins function to protect the genome from the consequences of DNA double strand breaks (DSBs). DSBs can occur as the result of external sources such as ionizing radiation or internal causes such as defects in the repair process. The inability to properly respond to DSBs can have catastrophic consequences for the cell, a fact highlighted by the prevalence of DSB repair proteins that are mutated in cancer (Lee and Paull 2005; Negrini et al. 2010). Consequently, most known organisms have evolved a comprehensive suite of mechanisms to repair DSBs appropriately, whether they have occurred by accident or design. A better understanding of DSB repair is therefore essential for understanding the mechanisms behind genome instability for the development of therapeutic approaches to prevent it.

One key player in the DNA damage response (DDR) to DSBs is the phosphatidylinositol 3-kinase (PI3K) related protein kinase ATM (Ataxia-telangiectasia-mutated). Mutation of ATM in humans can lead to Ataxia-telangiectasia (A-T), a rare autosomal recessive disorder, one of the hallmarks of which is a predisposition to developing cancer (Kurz and Lees-Miller 2004; Lavin 2008; Lavin et al. 2006; Lavin and Shiloh 1997). ATM is activated in response to DSBs and serves as a signal transducer, phosphorylating downstream targets to activate cell-cycle checkpoints, DNA damage response (DDR) proteins, and apoptosis pathways (Lavin 2008; Shiloh 2006). Human cells mutant for ATM are known to be defective in cell-cycle checkpoint activation, sensitive to DSB-inducing agents, and display chromosomal instability (Rotman and Shiloh 1998; Kojis et al. 1989; Pandita et al. 1995; Metcalfe et al. 1996; Xia et al. 1996).

*C. elegans* is a powerful model for investigation of the DNA repair and has a number of conserved DDR mechanisms (O’Neil and Rose 2006; Lemmens and Tijsterman 2011). The *C. elegans* ATM protein, ATM-1, is known to function in the DDR and DSB repair and its activity and functional interactions are conserved between worms and humans (Lemmens and Tijsterman 2011; Lee et al. 2010). Mutations in the *atm*-*1* gene result in animals that are sensitive to DSB inducing agents such as IR and, to a lesser extent, UV-C (Couteau and Zetka 2011; Garcia-Muse and Boulton 2005; Stergiou et al. 2007; Weidhaas et al. 2006). Genomic instability associated with mutations in ATM-1 has not, however, been documented. Furthermore ATM-1 appears structurally unique in that it is predicted to be a very small polypeptide when compared to other ATM homologs.

In this study we present the characterization of the *atm*-*1* locus in *C. elegans*. Using bioinformatic and molecular approaches we have revised the gene model for the *atm*-*1* and show that the full-length gene is a fusion of three predicted ORFs. We further describe the loss-of-function phenotype for this gene and identify a previously unrecognized mutator phenotype.

## Materials and methods

### Strains

*C. elegans* strains were maintained as described previously (Brenner 1974). Strains: VC2010 wild type, VC655 *brd*-*1*(*gk297*), FX5027 *atm*-*1*(*tm5027*), VC381 *atm*-*1*(*gk186*)*,* KR4941 *atm*-*1*(*gk186*) (outcrossed 10 times), BC2200 *eT1*(*III*)*/dpy*-*18*(*e364*)*;eT1*(*V*)*/unc*-*46*(*e177*), KR5060 *atm*-*1*(*gk186*)*; eT1*(*III*)*/dpy*-*18*(*e364*)*;eT1*(*V*)*/unc*-*46*(*e177*).

### Bioinformatic analysis

All nematode homology comparisons were performed using BLASTN and BLASTP (NCBI). Illumina-generated RNA-Seq data from an L1 synchronized cDNA library were analyzed for the *atm*-*1* transcript structure. Sequence reads were aligned to the *C. elegans* reference sequence WS200 using Bowtie (Langmead 2010) and Tophat (Trapnell et al. 2009). Default options were used, except insert size (-r) was set to 300 and the minimum intron size (-i) to 40. The alignment file was visualized using IGV (Integrated Genomics Viewer) (Robinson et al. 2011).

### cDNA library construction

A healthy mixed stage population of each strain was harvested for RNA extraction. The animals were pelleted by centrifugation and ~0.5 mL of packed worms resuspended in 2 mL of Trizol (Invitrogen, Catalog Number 15596-018). The worm/Trizol mix was vortexed, subjected to freeze–thaw, and incubated in a 37°C water bath. Nucleic acids were extracted with 1 mL chloroform followed by centrifugation and isolation of the supernatant. 1 mL isopropanol was added to the supernatant, mixed, and re-centrifugated. The nucleic acid pellet was washed with 0.5 mL RNAse-free 75% ethanol and allowed to dry and resuspended with DEPC-treated water. RNA quality was estimated on a 1% agarose gel. The nucleic acids were treated with DNase using the Fermentus DNase I, RNase-free kit and protocol (#EN0521). DNase was removed with phenol–chloroform followed by chloroform to isolate total RNA. RNA was precipitated overnight at three volumes of 100% ethanol to sample ratio with the addition of 0.3 M final concentration sodium acetate. Following centrifugation and removal of supernatant, the RNA pellet was washed with 0.5 mL 75% ethanol and resuspended in DEPC-treated water. cDNA was generated using the RevertAid H Minus M-MuLV Reverse Transcriptase kit from Fermentas using poly-dT primers (#EP0451). The quality and concentration of the total RNA and cDNA were measured using a NanoDrop spectrophotometer.

### Primer design and polymerase chain reaction (PCR)

Primers were designed using the Primer3 website interface http://frodo.wi.mit.edu/primer3/. cDNA PCRs were conducted using New England BioLabs Inc Phusion High-Fidelity DNA Polymerase to ensure high fidelity products for sequencing. Suggested Phusion protocol for PCR cycling was used: 98°C denaturing, 72°C elongation at 45 s/kb, primer specific annealing temperature (between 56–60°C) and cycled for 34 cycles. 10 mM stock dNTP solution was mixed from 100 mM stock solutions of each dNTP bought from Applied Biological Materials (abm) Inc. A 20 μL reaction consisted of 4 μL 5× HF buffer, 0.5 μL 10 mM dNTPs stock solution, 0.4 μL of each 10 μM primer stock, 100 ng cDNA, 0.1 μL Phusion polymerase, and 14.4 μL of double distilled water.

### Ionizing radiation sensitivity assay

For each strain, 30 adult animals were plated on 3 plates (10 worms per plate) and allowed to lay eggs for 2 h (giving approximately 50 eggs per plate.) These worms were synchronized to 1-day-old adults and subjected to different IR dosages. IR was applied by the TORREX 150D X-Ray Inspection System. After IR, worms were allowed to rest in 20°C for 20 h. 10 plates with 3 worms each were plated for each dose and a 4- to 5-h brood was collected. The number of unhatched eggs and adults was scored 24 and 72 h post brood collection, respectively. % survival was calculated by dividing the total number of adults by the total progeny (adults + unhatched eggs). Standard error was used for calculating statistical error.

### DAPI staining and microscopy

20–30 adult worms for each strain were picked onto a watch glass containing 5 μL of M9 buffer. 250 μL of 150 mM DAPI in 96% ethanol was added and allowed to evaporate in the dark at room temperature for 1–2 h. 500 μL of M9 was used to destain the worms for 2 h at room temperature. Worms were mounted 3% agarose pads. Diakinetic chromosomes were observed in oocytes located before the spermatheca using a Zeiss Axioscope fluorescent microscope with 40× objective using OpenLab software.

### Quantifying genomic instability

20 *atm*-*1*(*gk186*) L4 worms were individually plated and the subsequent progeny scored for total progeny and the presence of males. A single L4 worm from each line was plated to propagate the line. This was repeated for 20 generations. Total brood was scored and the presences of males (>10% of the total population) were recorded.

## Results

### *C*. *elegans* ATM-1 has a conserved kinase domain but lacks upstream sequences

**Fig. 1:**
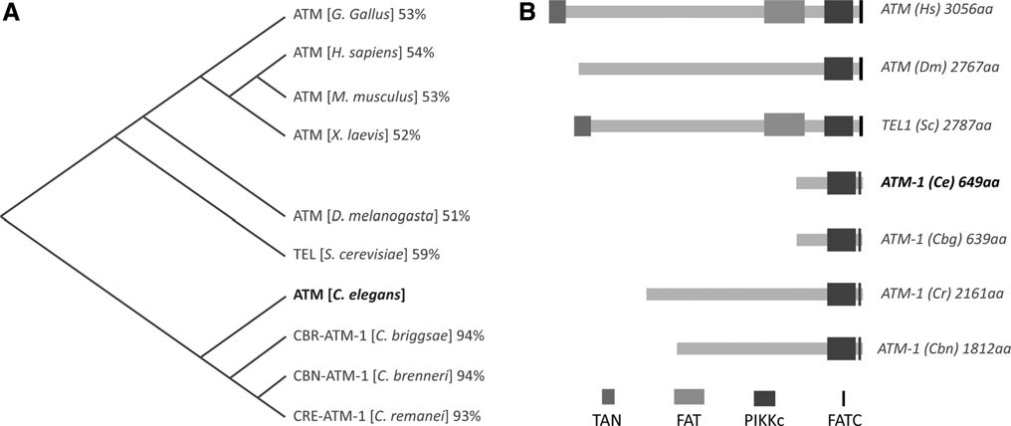
*C. elegans* ATM-1 is much smaller than other ATM orthologs including those in closely related nematode species. **a** Distant matrix tree of ATM orthologs based on conservation of the kinase domain. Percentage similarity to *C. elegans* is indicated. **b** Schematic representation of protein structure of human ATM orthologs from other species. *TAN* Tel1/ATM N-Terminal motif, *FAT* FRAP–ATM–TRAP domain, *PIK* phosphatidylinositol 3-kinase, *FATC* C-Terminal FAT domain

Human ATM is a large protein (3056aa) with a highly conserved kinase domain and a large, poorly conserved, N-terminal region (Fig. 1a; data not shown). In contrast *C. elegans* ATM-1 (*Ce*-ATM-1) is predicted to be a relatively small 649AA polypeptide similar in structure to the C-terminal portion of ATM, having the conserved kinase domain and FATC domains (Fig. 1b). ATM-1 is, however, lacking domains known to be present in other ATM orthologs, including the FAT and TAN domains that are typically upstream of the kinase domain (Bosotti et al. 2000; Seidel et al. 2008) (Fig. 1b). The discrepancies between *Ce*-ATM-1 and other ATM orthologs led us to investigate its structure in more detail.

### Cross-species analysis of nematode genomes suggests an extended *C. elegans* ATM-1 structure

**Fig. 2:**
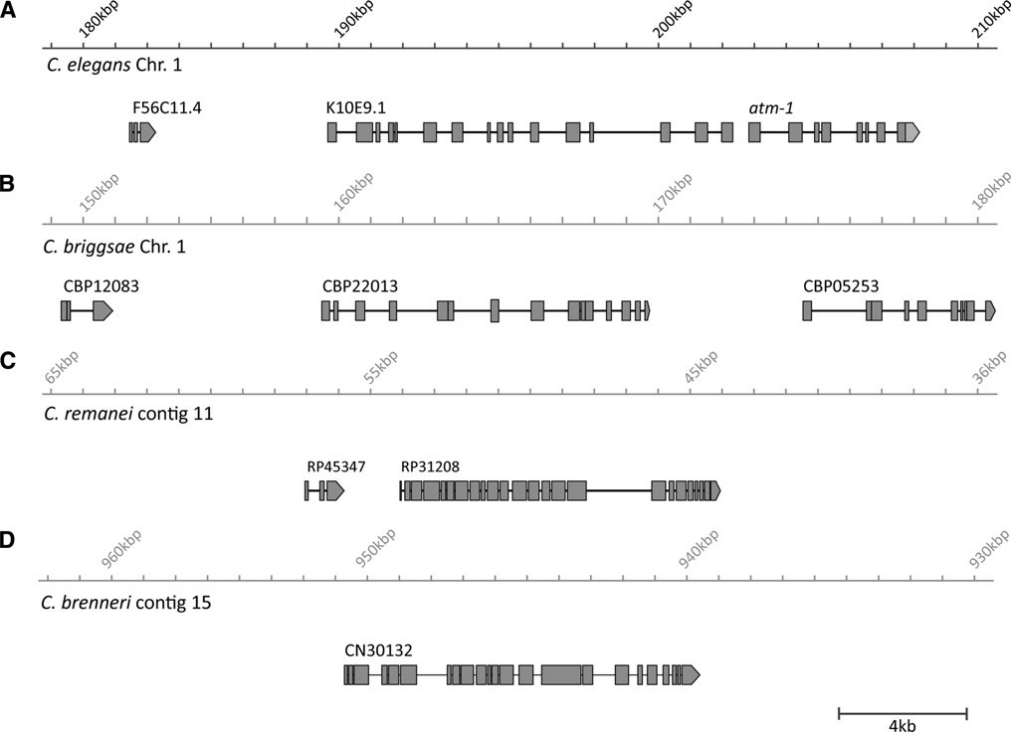
Evidence for an extended *atm*-*1* transcript in closely related nematode species. **a**–**d** Schematic representations of gene predictions in the genomic region of *atm*-*1* in four nematode genomes. **a** In *C. elegans* the region contains three gene predictions. **b** *C. briggsae* also contains three gene predictions in the equivalent region. **c** In *C. remanei* two gene predictions are annotated, the genes corresponding to K10E9.1 and *atm*-*1* are represented as a single gene, RP31208. **d** A single gene prediction is annotated in*. C. brenneri*. Figure is to scale

We used BLASTP to compare the predicted sequences of ATM-1 in closely related *Caenorhabditis* species; *C. briggsae* (*Cbg*), *C. remanei* (*Cr*) and *C. brenneri* (*Cbn*) (Fig. 2). Interestingly the predicted sizes of *Cbn*-ATM-1 (1812aa) and *Cr*-ATM-1 (2161aa) are significantly larger than both *Ce*-ATM-1 (649aa) and *Cbg*-ATM-1 (639aa) (Fig. 1b). Furthermore, BLASTP comparison of *Cbn*-ATM-1 revealed matches not only with *Ce*-ATM-1 but also with two gene predictions located further upstream, K10E9.1 and F56C11.4 (Fig. 2a). These three gene predictions might therefore comprise a single, extended, *atm*-*1* transcript. Likewise the equivalent genomic region in *C. briggase* includes three distinct gene predictions (Fig. 2b) while in *C. remanei* there are only two (Fig. 1c). The gene order is maintained between these nematode species. It is likely that the *Cbr*-*atm*-*1* and *Cr*-*atm*-*1* gene models are also misannotated.

### Transcriptome and EST analysis support the extended gene structure

**Fig. 3:**
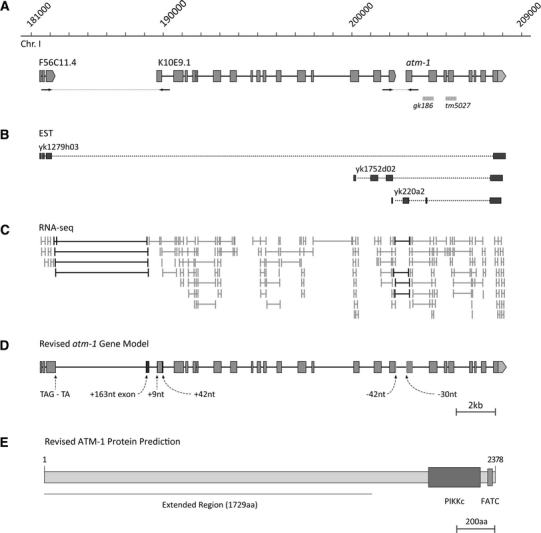
Revised *C. elegans atm*-*1* gene structure. **a** A schematic showing the original gene model predictions for the genomic region of *atm*-*1*. Position of primer pairs used to confirm transcript junctions by PCR is shown below the model (not to scale). Regions deleted in the knockout alleles *gk186* and *tm5027* are depicted as *dashed bars*. **b** Schematic of ESTs confirming the extended *atm*-*1* gene model. **c** Representation of RNA-seq mapping data. Sequence tags confirming merging between gene predictions are highlighted in *black*. **d** Schematic showing the revised transcript structure for *Ce*-*atm*-*1*. Changes to exonic structure are listed below the figure. **e** Schematic of the extended ATM-1 protein. Figure is to scale

Three annotated expressed sequence tags (ESTs) from the Kohara group (Tabara et al. 1996) provided evidence for an extended *atm*-*1* transcript. The ESTs *yk1752d02* and *yk220a2* both span the junction between the K10E9.1 and *atm*-*1* gene predictions while a single EST, *yk1279h03*, links F56C11.4 to the same transcript (Fig. 2b). To obtain further evidence of an extended *Ce*-*atm*-*1* transcript we used available deep RNA-seq data to map transcript sequences to F56C11.4, K10E9.1 and *atm*-*1* (Our unpublished data). From the data we identified four tags that spanned the junction between F56C11.6 and K10E9.1, and a further four tags that spanned the junction between K10E9.1 and *atm*-*1* (Fig. 3c)*.* Thus, we conclude that F56C11.4, K10E9.1 and *atm*-*1* produce a single transcript.

### The gene predictions F56C11.4, K10E9.1, and Y48G1BL.2 (*atm*-*1)* produce a single mRNA transcript

To independently confirm the structure of the extended *atm*-*1* transcript a wild-type (N2) cDNA library was probed with two sets of PCR primers designed to amplify the predicted junctions between adjacent open reading frames (Arrows in Fig. 3a). Both PCR assays amplified a sequence of expected size from the WT cDNA (data not shown)*.* Full length sequencing of the cDNA transcript confirmed the extended structure for *atm*-*1* (Fig. 3d). The majority of the transcript sequence aligned with the predicted gene models as expected. However, a previously unidentified 5′ splice site disrupts the annotated stop codon of F56C11.4. The terminal exon of F56C11.4 splices to a previously unknown exon of 163nt located 433nt upstream of the predicted first exon of K10E9.1. Exon 1 of K10E9.1 has additional exonic sequences added at both 5′ (9nt) and 3′ (42nt). Finally an alternative 5′ splice site results in removal of 42nt from the end of the last predicted exon of K10E9.1 and a 3′ splice site removes 30nt from the beginning of the first predicted exon of *atm*-*1*, joining these two ORF’s. All transcript modifications were also present in the RNA-seq data (data not shown). The revised *atm*-*1* gene model produces an ORF of 7,137nt that is predicted to encode a protein of 2,378AA (Fig. 3e). Domain analysis of the revised ATM-1 protein prediction did not, however, reveal any additional conserved domains beyond the kinase and FATC domains (Fig. 3e).

### Transcript analysis of the *gk186* gene reveals two potential transcripts

Two knockout mutations of *atm*-*1* are available, *gk186* and *tm5027*. *atm*-*1*(*tm5027*) is a 513 bp deletion that disrupts exon 23 and deletes exon 24, resulting in 4 missense amino acids and a premature stop in exon 23 (Fig. 3a). The *tm5027* mutant transcript results in a truncated protein lacking the kinase domain of the protein and is therefore expected to be null (Figure S1). *atm*-*1*(*gk186*) is a 548 bp deletion that removes the 3′ intron splice site for the 22nd exon of the revised *atm*-*1* gene model (Fig. 3a), resulting in the splicing of exons 21–23 (Figure S1) (Garcia-Muse and Boulton 2005; Stergiou et al. 2007). The mutant transcript is predicted to produce a frame-shift mutation resulting in a truncated protein lacking the kinase domain of the protein. However, a second mRNA species was present in cDNA from *gk186* animals (Figure S1). Sequence analysis of PCR fragments from *gk186* revealed that an alternative splice acceptor allows splicing of exon 24, generating a transcript that maintains the kinase domain (Figure S1; data not shown).

### Animals carrying either *gk186 ortm5027* are sensitive to IR

**Fig. 4:**
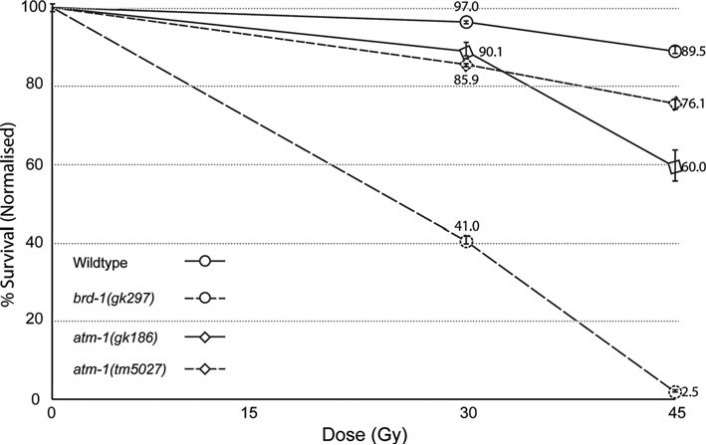
*atm*-*1* mutants are sensitive to IR. Graph showing the ionizing radiation (IR) sensitivity of wild type, *brd*-*1* mutant, and *atm*-*1* mutant worms. Survival percentage is calculated by dividing the number of adults by the sum of arrested embryos and adults multiplied by 100. *Error bars* indicate the standard error

In order to determine the radiation sensitivity of the mutant animals, we treated animals from strains carrying *gk186 or* *tm5027* with ionizing radiation (IR). We measured the survival frequency of the progeny of parental hermaphrodites exposed to IR. Wild type and mutants in a known IR-sensitivity gene, *brd*-*1*(*gk297*), were used as controls. The results are shown in Fig. 4. Approximately 90% of the wild-type F1’s hatched and survived at the highest dose tested (45 Gy), compared to 2.5% F1 survival in the *brd*-*1* mutant animals. The *atm*-*1* mutant animals had an intermediate survival frequency that was significantly different than the wild type. Of the two *atm*-*1* mutants, *gk186* was significantly more sensitive at the higher dose than *tm5027*. Thus, the alternative transcript produced in *gk186*, which contains the kinase domain does not contribute to IR resistance. The knockout allele, *tm5027* deletes the kinase domain and can be assumed to exhibit the loss-of-function phenotype. Thus, we conclude that the data shown in Fig. 4 represent the null phenotype for *atm*-*1*.

### *atm*-*1* mutant animals display the hallmarks of a mutator

Morphologically, *atm*-*1* mutant animals appear superficially wild type. In maintaining these strains, however, we observed variation in the number and appearance of the offspring of specific individuals. Brood analysis of both mutant strains revealed erratic brood sizes. Some hermaphrodites were sterile and some produced arrested embryos. Embryonic arrest varied between 1 and 50% (Table 1). The appearance of a high incidence of male (Him) phenotype was also consistently observed. These male-producing lines could be maintained by picking individual hermaphrodites indicating that the high incidence of male (Him) phenotype was a heritable trait. In *C. elegans*, sterility and Him phenotypes are both indicators of genomic instability (Ahmed and Hodgkin 2000; Herman et al. 1982). It is likely that such phenotypes are arising due to the mutagenic consequence of persistent DSBs in the absence of ATM-1 function.

**Table 1:** Fertility and viability analysis of *atm*-*1*(*gk186*) and *atm*-*1*(*tm5027*) animals

Plate	Wild type (N2)			*atm*-*1* (*gk186*)			*atm*-*1* (*tm5027*)		
#	Adult	Emb	Total	Adult	Emb	Total	Adult	Emb	Total
1	223	2	225	202	1	203	174	3	177
2	239	0	239	98	70	168	119	9	128
3	309	1	310	189	2	191	65	0	65
4	259	2	261	101	83	185	188	13	201
5	322	1	323	103	88	191	228	2	231
6	160	3	163	205	1	207	96	99	196
7	229	2	231	174	3	177	109	50	159
8	246	1	247	163	1	164	^a^	^a^	^a^
9	300	1	301	^a^	^a^	^a^	^a^	^a^	^a^
10	271	0	271	^a^	^a^	^a^	^a^	^a^	^a^
Total	2,558	13	2,571	1,235	249	1,486	979	176	1,157
Average	256	1	257	154	31	186	140	25	165

^a^F1 animal was sterile and produced no progeny

### Quantifying independent *atm*-*1*(*gk186*) lines indicate inherited genomic instability

**Fig. 5:**
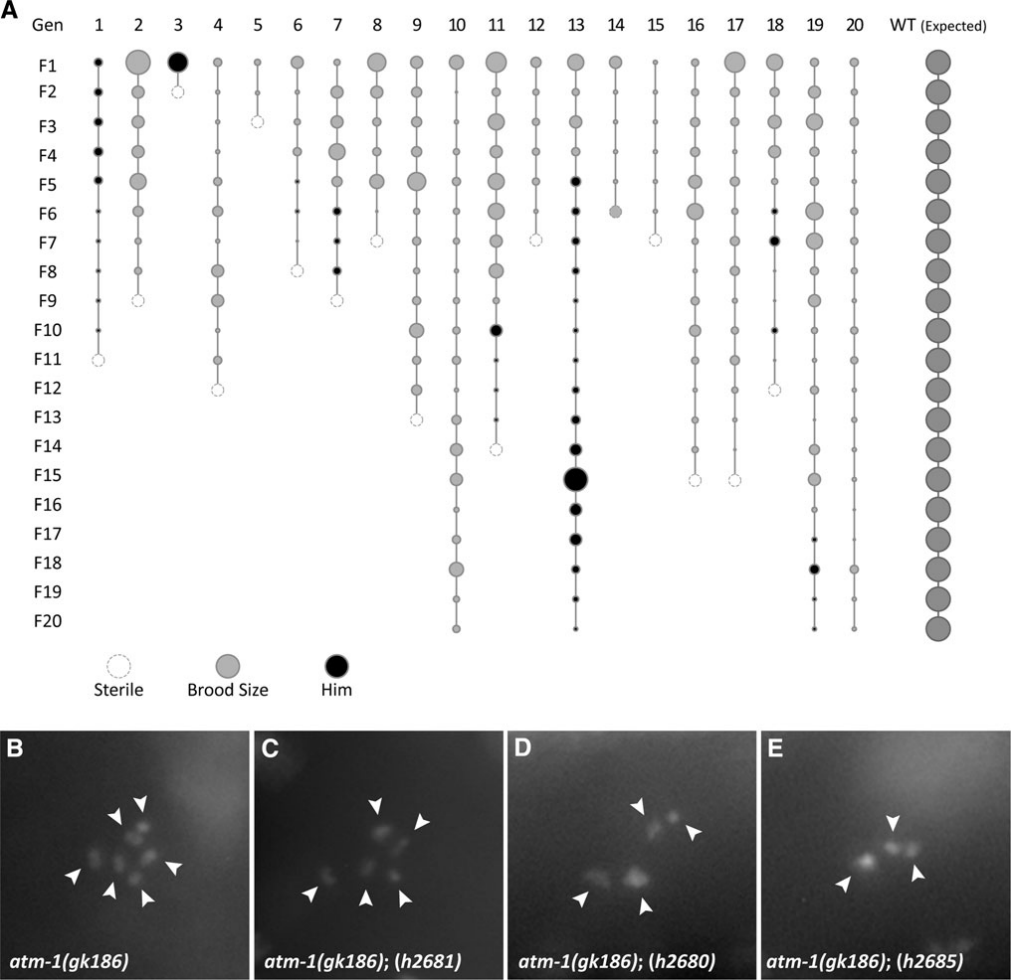
*atm*-*1*(*gk186*) animals display pleiotrophic phenotypes associated with genome instability. **a** 20 individual animals were isolated and their progeny examined over 20 subsequent generations for brood size, viability, a Him phenotype and sterility. The brood size of each animal is directly proportional to *size of the circle*. **b**–**e** DAPI staining of diakinetic chromosomes (*arrowheads*) in *atm*-*1*(*gk186*) animals with a dominant Him phenotype. **b** *atm*-*1*(*gk186*) mutant animals have the expected compliment of six chromosomes. **c** *atm*-*1*(*gk186*)*;*(*h2681*) animals have five chromosomes. **d** *atm*-*1*(*gk186*)*;*(*h2680*) animals have four chromosomes. **c** *atm*-*1*(*gk186*)*;*(*h2685*) animals have three chromosomes

To further document the variability in the phenotype of *atm*-*1* mutants, we examined the viability of *atm*-*1*(*gk186*) animals over 20 generations. 20 L4 *atm*-*1*(*gk186*) worms were plated individually and scored for viability, brood size, and the presence of males. Each subsequent generation was established by selecting a single L4 offspring at random. This process was repeated for 20 generations and the results are summarized in Fig. 5a and Table S1. Over the course of the experiment 8 of the 20 individual lines acquired Him mutations. 16 of 20 lines became sterile; including several that had first become Him. The progeny numbers for the different lines also fluctuated unpredictably over the 20 generations (Fig. 5a; Table S1). Together these results point to a model in which heritable mutations that confer a high incidence of male and sterile phenotypes, as well as mutations that result in loss of viability are generated in *atm*-*1* mutant animals.

### *atm*-*1*-derived dominant him strains have a reduced chromosome number

We reasoned that since ATM is also known to function in telomere maintenance the Him phenotype observed might also be due to chromosome fusions (Ahmed and Hodgkin 2000; Herman et al. 1982; Herman and Kari 1989). Five independent *atm*-*1*(*gk186*) lines that became Him were isolated and their chromosome complement assessed by visualizing diakinetic chromosomes in their ooctyes. Normal wild-type animals and freshly outcrossed *atm*-*1*(*gk186*) mutants have the expected complement of six pairs of chromosomes, seen as distinct spots when visualized using DAPI staining (Fig. 5b; data not shown). In all of the *atm*-*1*(*gk186*) Him strains examined a reduced number of diakinetic chromosomes were observed (Fig. 5c–e; Table 2). A maximum of five chromosomes was seen in these strains though animals with only four or three pairs were also observed (Fig. 5). This indicated that multiple fusions were occurring with no loss of essential genetic material. The fact that less than five chromosome pairs were seen in viable Him animals also indicated that chromosome fusions were not specific to the X chromosome. This is consistent with the interpretation that loss of function of *atm*-*1* leads to chromosome rearrangement.

**Table 2:** Diakinetic chromosome count in *atm*-*1*(*gk186*) derived Him strains visualized by DAPI staining

Strain	# of Diakinetic chromosomes
WT	6
*atm*-*1* (*gk186*)	6
*atm*-*1* (*gk186*)*; h2680*	4
*atm*-*1* (*gk186*)*; h2681*	5
*atm*-*1* (*gk186*)*; h2682*	5
*atm*-*1* (*gk186*)*; h2683*	5
*atm*-*1* (*gk186*)*; h2684*	5
*atm*-*1* (*gk186*)*; h2685*	3

### Mutations produced in an *atm*-*1*(*gk186*) background can be captured using a translocation balancer

Chromosome fusions appear to be one of the consequences of loss of *atm*-*1* function. We have also shown that fusions are likely to be occurring between autosomes also. However, the sterility and reduced brood numbers observed suggest that mutations resulting in the loss of essential genetic material are also occurring. This indicates that the mutational spectrum of *atm*-*1* is not limited to viable chromosome fusions. To explore this further we used the *eT1* balancer system (Rosenbluth and Baillie 1981) to capture mutational events induced in an *atm*-*1*(*gk186*) background. *eT1* is a stable reciprocal translocation of the *C. elegans* chromosomes (Chr) III and V. Recombination is effectively suppressed in regions of Chr III and Chr V involved in the translocation and mutations present in the balanced regions are maintained heterozygously in these strains (Rosenbluth and Baillie 1981; Johnsen and Baillie 1991). Visible markers are used to follow the non-*eT1* chromosomes. Loss of these markers in the viable progeny of this strain is an indication that an essential mutation has occurred within the balanced region. 1,139 *atm*-*1*(*gk186*)*; eT1* animals were plated and their progeny were screened for the absence of the visible markers, indicating that a lethal mutation had occurred. 11 independent lines harboring mutations obtained (Table S2) a number of mutational events equivalent to a 0.97% forward mutational frequency for *atm*-*1*(*gk186*), compared to 0.06% for the *eT1* balancer system alone (Rosenbluth and Baillie 1981). This is similar mutation rate to that of *dog*-*1* (0.9%), a known mutator that is required to maintain genome stability by resolving potentially toxic G-track secondary structures that form during replication (Cheung et al. 2002). The results show that in addition to heritable male-producing strains, non-Him-associated lethal mutations occur and can be isolated and stably maintained.

## Discussion

We describe here the structure of the *atm*-*1* gene in *C. elegans* and its loss of function phenotype. Revealing the structure and function of ATM is an important step in understanding the human disease A-T specifically and the DNA damage response in general. Such functional studies require a solid platform in a model organism. Here, we establish that *C. elegans* can be used as a model for the study of ATM by correcting the annotated gene structure of *atm*-*1* and documenting the phenotypes arising in deletion mutants of this gene. Here, we have shown that the *atm*-*1* gene model reported in wormbase (WS228) is incomplete, most notably lacking the large N-terminal region present in other ATM homologs.

We have shown that the publically available *atm*-*1* gene model was incomplete as it lacked a large N-terminal region present in other ATM homologs (Fig. 1b). The revised model merges the *atm*-*1* gene prediction with the upstream gene predictions *K10E9.1* and *F56C11.4* leading to a protein structure that is more in keeping with other ATM homologs. In addition to the size difference, we have identified previously unpredicted exonic sequences in the *atm*-*1* transcript, refining the model further (Fig. 3d).

Regarding the nature of the *gk186* mutation*,* one recent report identified a transcript that utilizes a cryptic splice site located in the exon affected by the deletion (Stergiou et al. 2007). Based on this translation, the authors predicted an ATM-1 protein lacking the kinase domain and suggested that *atm*-*1*(*gk186*) is a null mutation. In addition to the reported transcript (*atm*-*1.a* Figure S1) we have also identified a second transcript, *atm*-*1*(*gk186*)*.b*. This transcript utilizes two alternative splice sites to produce an in-frame translation resulting in an internal deletion of 112AA. The translated protein preserves the PI3K kinase domain and might therefore retain some kinase function. Two independently derived mutant strains were sensitive to treatment with IR. In addition, both strains also display the hallmarks of genomic instability. We conclude that our results represent the null phenotype for the *atm*-*1* gene and that the alternative transcript observed in the *gk186*-containing mutant is either non-functional or functional at an insignificantly low level.

In contrast to previous reports (Bailly et al. 2010), we find that *atm*-*1*(*gk186*) *mutants* are sensitive to IR. We have confirmed the IR sensitivity with the *tm5027* allele. Experimental factors such as the brood size collected and the rest period might account for this discrepancy. The effect of IR on *atm*-*1* mutants is not severe when compared to *brd*-*1*(*gk99*) mutants. *brd*-*1* forms a heterodimer with *brc*-*1* and is required for all homologous recombination (HR) repair of DSBs (Boulton et al. 2004). The fact that the *atm*-*1* mutants are only partially sensitive to IR may indicate functional redundancy in repair of DSBs as proposed by Bailly et al. (2010).

We successfully used *eT1* to capture lethal mutations generated in an *atm*-*1*-deficient background. The forward mutation frequency for *atm*-*1*(*gk186*) in this experiment was calculated to be 0.96%, which is similar to that reported for *dog*-*1* (Cheung et al. 2002), and comparable to low dosages of other mutagens [1.5% for 0.004 M EMS, 0.96% for 500 Roentgens of gamma radiation, and 1.58% for 0.11% formaldehyde treatment (Rosenbluth and Baillie 1981)]. Since *eT1* covers approximately one-sixth of the genome, this translates into a 7% forward mutation rate for the whole gene in the absence of ATM function. Thus, although not an essential gene, *atm*-*1* clearly has a significant role in maintaining genomic stability.

In human A-T cell lines, reduced telomere length, increased rate of telomere shortening, and telomere fusions are observed (Metcalfe et al. 1996; Pandita et al. 1995; Xia et al. 1996). In both fission and budding yeast, a role for preventing telomere fusions and chromosome translocations has also been documented (Lee et al. 2008; Naito et al. 1998). No function for ATM-1 in telomere maintenance has been reported in *C. elegans* until now. Here, we have shown that *atm*-*1* mutants can give rise to a heritable Him phenotype. Him phenotypes are known to arise in animals where X:autosome rearrangements and X:autosome telomeric fusions have occurred (Ahmed and Hodgkin 2000; Herman and Kari 1989; Herman et al. 1982). Ahmed and Hodgkin, have identified and characterized a mutant (*mrt*-*2*) that results in X:autosome fusions. *mrt*-*2* encodes a highly conserved DNA-damage checkpoint protein homologous to RAD1. Him mutants arising in a *mrt*-*2* genetic background also have a reduced number of diakinetic chromosomes (Ahmed and Hodgkin 2000) consistent with what we have observed in *atm*-*1*-derived Him animals. We conclude that these mutations are also likely to be X:autosome telomere fusions. The observation that less than five pairs of chromosomes are observed in some of the Him animals indicates that chromosomal fusions are not restricted to the X chromosome.

Reduced viability, a Him phenotype, and sterility are all indicators of genomic instability. We have documented independent lines of *atm*-*1* mutants that display all of these phenotypes. That these mutations lead to inviability indicates that essential genetic material has been disrupted. Our characterization of the mutant ATM phenotype in *C. elegans* indicates that a range of chromosomal instabilities occurs that reflect a rich-mutagenic spectrum, not limited to chromosomal fusion events. In this regard, *C. elegans* provides a valuable model for the study of ATM function in a living animal and its role in preventing chromosomal abnormalities, providing insight to the function of *atm*-*1* in promoting genomic stability.

## Conclusion

We provide a revised structure for the *C. elegans atm*-*1* gene that merges three gene annotations and reinterprets the splicing structure of the gene. The reinterpreted *atm*-*1*-coding region includes the ORFs F56C11.4, K10E9.1, and the current *atm*-*1* gene prediction. Transcript analysis indicates that there is a single transcript produced that is required for response to ionizing radiation and maintenance of genomic stability. Lethal mutational events were isolated using the *eT1* balancer system. Future work on *atm*-*1* in *C. elegans* would provide insight into the DDR and intracellular signalling and control. *C. elegans* and *atm*-*1* can possibly be used to understand the structure and function of telomere fusions with regard to tumourigenesis. Perhaps the most significant contribution to understanding *atm*-*1,* and ATM in general, would be discovering novel therapeutic targets for ATM-driven cancers. ATM interactions for synthetic lethality could lead to targeted therapy for appropriate cancers. Research in these areas would be greatly facilitated through *C. elegans* because it is amendable to high throughput, cost-effective methods of study. Our results establish *C. elegans* as an excellent model for the study of ATM function.

## Electronic supplementary material

Below is the link to the electronic supplementary material.

Supplementary material 1 (DOCX 6031 kb)
