# Supplementary material for: The atm-1 gene is required for genome stability in Caenorhabditis elegans
Source: Mol Genet Genomics. 2012 Feb 18;287(4):325–35. doi: 10.1007/s00438-012-0681-0 (PMC3313021; doi:10.1007/s00438-012-0681-0)
Supplement: Supplementary file 1 — Supplementary material 1 (DOCX 6031 kb) [file 438_2012_681_MOESM1_ESM.docx]

**The *atm-1* gene is required for genome stability in *Caenorhabditis elegans***

**Martin R. Jones^1*^, Jim Chin Huang^1*^, Shu Yi Chua^2^, David L. Baillie^2^ and Ann M. Rose^1+^**

^1^Department of Medical Genetics, University of British Columbia, 419 – 2125 East Mall, Vancouver, BC, V6T 1Z4, Canada

^2^Molecular Biology and Biochemistry, Simon Fraser University, Burnaby, B.C. V5A 1S6

*These authors contributed equally to this work.

**^+^Corresponding author: 604 822-5467**

**Email:** [**Ann.Rose@ubc.ca**](mailto:Ann.Rose@ubc.ca) **(A.M. Rose)**

**MR Jones** [**mjones@gene.nce.ubc.ca**](mailto:mjones@gene.nce.ubc.ca)

**CJ Huang** [**JHuang02@gmail.com**](mailto:JHuang02@gmail.com)

**SY Chua** [**sychua@sfu.ca**](mailto:sychua@sfu.ca)

**DL Baillie** [**Baillie@sfu.ca**](mailto:Baillie@sfu.ca)

­
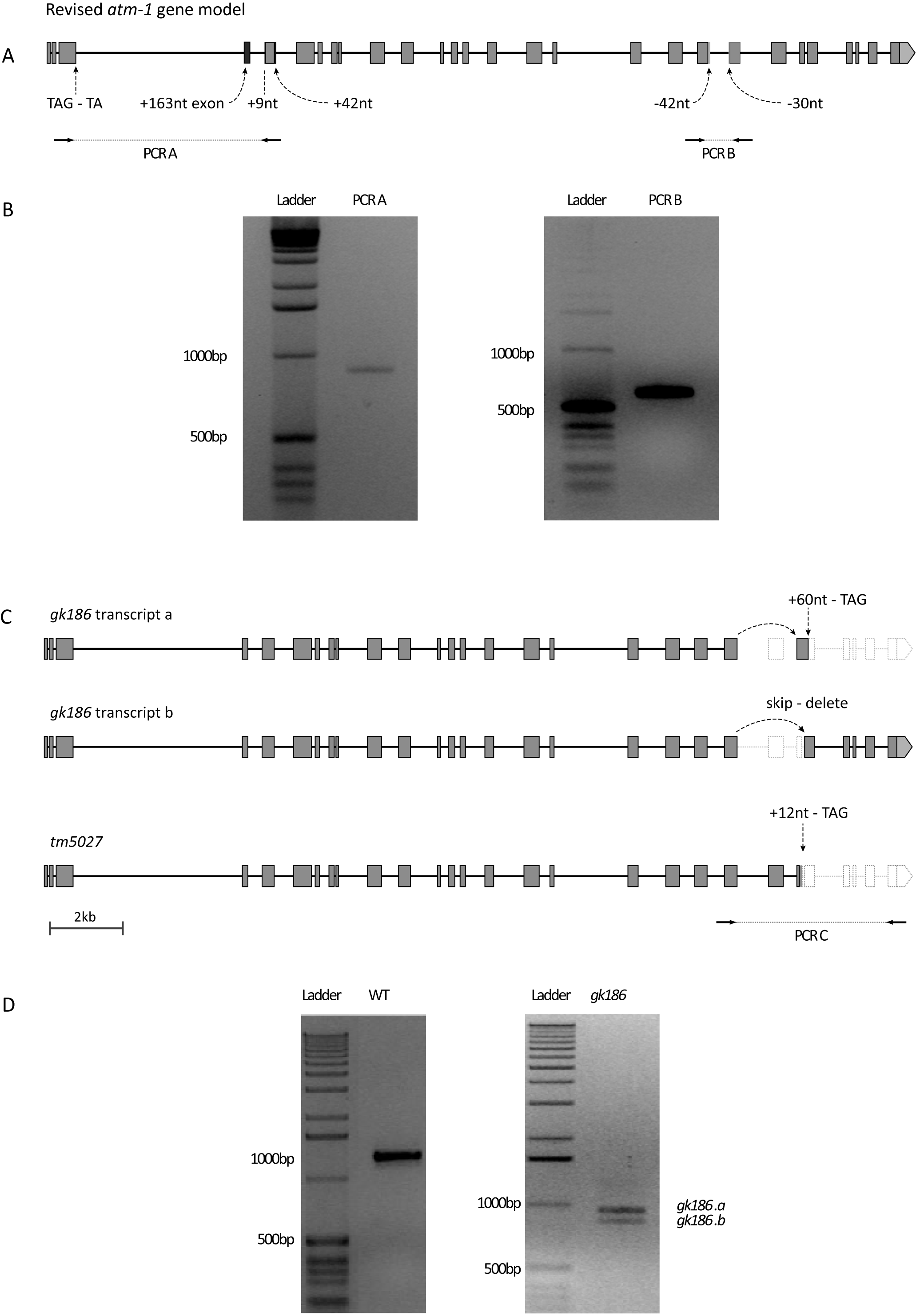


**Figure S1. *atm-1* Mutant Transcripts.** A. Revised *atm-1* transcript structure. B. PCR gels of ORF junction in WT cDNA. C. Predicted transcripts in *atm-1* mutant alleles D. PCR gels showing two bands in *gk168* mutant cDNA. See text for details. Figure is to scale.

Table S1. Raw data from the 20-generation stability analysis

| Line | 1 | 2 | 3 | 4 | 5 | 6 | 7 | 8 | 9 | 10 | 11 | 12 | 13 | 14 | 15 | 16 | 17 | 18 | 19 | 20 |
| --- | --- | --- | --- | --- | --- | --- | --- | --- | --- | --- | --- | --- | --- | --- | --- | --- | --- | --- | --- | --- |
| F1 | **108** | 282 | **245** | 110 | 63 | 126 | 79 | 246 | 136 | 187 | 262 | 118 | 225 | 141 | 16 | 84 | 240 | 212 | 108 | 91 |
| F2 | **116** | 144 | - | 49 | 1 | 37 | 138 | 133 | 77 | 6 | 116 | 58 | 83 | 16 | 30 | 83 | 125 | 111 | 151 | 61 |
| F3 | **142** | 147 |  | 41 | - | 60 | 144 | 89 | 125 | 40 | 187 | 79 | 146 | 12 | 32 | 128 | 95 | 178 | 189 | 107 |
| F4 | **144** | 160 |  | 24 |  | 80 | 202 | 78 | 130 | 59 | 158 | 74 | 116 | 25 | 29 | 130 | 44 | 152 | 121 | 31 |
| F5 | **167** | 218 |  | 78 |  | 37 | 126 | 172 | 230 | 98 | 206 | 84 | **131** | 38 | 24 | 159 | 125 | 67 | 95 | 44 |
| F6 | **4** | 113 |  | 91 |  | 30 | 107 | 10 | 61 | 38 | 206 | 20 | **107** | - | 14 | 203 | 57 | **60** | 232 | 86 |
| F7 | **37** | 69 |  | 48 |  | 6 | **61** | - | 114 | 37 | 154 | - | **96** |  | - | 51 | 119 | **83** | 187 | 85 |
| F8 | **11** | 87 |  | 135 |  | - | **79** |  | 37 | 31 | 187 |  | **77** |  |  | 43 | 115 | **5** | 117 | 60 |
| F9 | **12** | - |  | 160 |  |  | - |  | 103 | 45 | 59 |  | **35** |  |  | 94 | 37 | **8** | 144 | 40 |
| F10 | **5** |  |  | 49 |  |  |  |  | 183 | 81 | **138** |  | **38** |  |  | 135 | 68 | **58** | 61 | 81 |
| F11 | - |  |  | 97 |  |  |  |  | 88 | 107 | **18** |  | **31** |  |  | 83 | 51 | **7** | 37 | 74 |
| F12 |  |  |  | - |  |  |  |  | 113 | 43 | **20** |  | **93** |  |  | 23 | 60 | - | 104 | 40 |
| F13 |  |  |  |  |  |  |  |  | - | 113 | **23** |  | **123** |  |  | 61 | 20 |  | 4 | 50 |
| F14 |  |  |  |  |  |  |  |  |  | 148 | - |  | **168** |  |  | 67 | 3 |  | 90 | 33 |
| F15 |  |  |  |  |  |  |  |  |  | 166 |  |  | **298** |  |  | - | - |  | 155 | 34 |
| F16 |  |  |  |  |  |  |  |  |  | 41 |  |  | **158** |  |  |  |  |  | 47 | 14 |
| F17 |  |  |  |  |  |  |  |  |  | 107 |  |  | **165** |  |  |  |  |  | **46** | 9 |
| F18 |  |  |  |  |  |  |  |  |  | 181 |  |  | **111** |  |  |  |  |  | **82** | 100 |
| F19 |  |  |  |  |  |  |  |  |  | 53 |  |  | **58** |  |  |  |  |  | **24** | 33 |
| F20 |  |  |  |  |  |  |  |  |  | 77 |  |  | **1** |  |  |  |  |  | **29** | 24 |

Numbers underlined and in bold indicate populations with a high proportion of males (>10%).

Table S2. *atm-1(gk186)* derived lethal mutations.

| _KR5072_ |  |  | *_atm-1 (gk186) ; eT1 [unc36] / dpy-18 (e364) III ; unc-46 (e177) V let-(h2809)_* |
| --- | --- | --- | --- |
| _KR5073_ |  |  | *_atm-1 (gk186) ; eT1 [unc36] / dpy-18 (e364) III ; unc-46 (e177) V let-(h2810)_* |
| _KR5074_ |  |  | *_atm-1 (gk186) ; eT1 [unc36] / dpy-18 (e364) III ; unc-46 (e177) V let-(h2811)_* |
| _KR5075_ |  |  | *_atm-1 (gk186) ; eT1 [unc36] / dpy-18 (e364) III ; unc-46 (e177) V let-(h2812)_* |
| _KR5076_ |  |  | *_atm-1 (gk186) ; eT1 [unc36] / dpy-18 (e364) III ; unc-46 (e177) V let-(h2813)_* |
| _KR5077_ |  |  | *_atm-1 (gk186) ; eT1 [unc36] / dpy-18 (e364) III ; unc-46 (e177) V let-(h2814)_* |
| _KR5078_ |  |  | *_atm-1 (gk186) ; eT1 [unc36] / dpy-18 (e364) III ; unc-46 (e177) V let-(h2815)_* |
| _KR5079_ |  |  | *_atm-1 (gk186) ; eT1 [unc36] / dpy-18 (e364) III ; unc-46 (e177) V let-(h2816)_* |
| _KR5080_ |  |  | *_atm-1 (gk186) ; eT1 [unc36] / dpy-18 (e364) III ; unc-46 (e177) V let-(h2817)_* |
| _KR5081_ |  |  | *_atm-1 (gk186) ; eT1 [unc36] / dpy-18 (e364) III ; unc-46 (e177) V let-(h2818)_* |
| _KR5082_ |  |  | *_atm-1 (gk186) ; eT1 [unc36] / dpy-18 (e364) III ; unc-46 (e177) V let-(h2819)_* |

File S1. Revised ATM-1 protein prediction

>Revised *atm-1* transcript

ATGAGCGTTACTCAGCTAAAAAATCTGAAACACGCGATAGCTCAGCTTCTGGAGTGGGATGGAACGAAAACGGCGAGAAAGAAAATCGTCGACGAGGTGGTTCTTCTCTATCACGCTCTGGGAGCGGAGGCATTAAGTGAGGATAACCAGGAGATATATGATTTGTATGATTTATCTGCACGTATATTCAACTTAGCTAAGAAAGAAATCGAAGAAGCCAACCAACAATTCGAAAAAGAACGAAAAAAGGGCACAAGACGAAGCGAAAAACCAGTCCCAACTCCACTTTTCGAGCTATCAATACAACATTTAAAACGTTGCTGTCAGCAGGGAATCGATCACAATCAGGTGCCATGGATAGCATATTGTCTGAAACTACTGGAATTTCCAATCACAATCACCGAAAAATCGATCGAAAACGAGATTTCCAACGTGCTCCTATTGAGCTCCAACGCCTCACAGCTCCATTGGGCCGAGCATGCTCATTTGAGCAGCTTATGGAAATGGATTTGGAGCCGTGTCGAGACCGCCGATATTGGGGCACTCGCCATGAGAAATTATATGGAATTGGCGGCGAACTTGCTGGAAAACGTGGATTACGTGGTTTTCGAGAAGTCGCCTATTGACCTGATGGCGAAAGTGATGGGAACACTGAAGAAAAGTGTGGAAATGGGAAATCCCAAAGAATATTGGTGTCATTACCGAAAATTCTCGGTCGTCCACATTCTCTCATATATCGTACATCGTTGGGGTCTCGAAGCGAGAGATTTCATTCTTCGTGAGATTTTCGAGCTCACTGGTAGTTTGTCCAACTTGATATCAGTGGCTCATAAGGATGGAGAACAGTCCAAGAAATGCATAATGCGTTTGATTGATGATCTCGTGAAGCTCGCCATGATCGAAACCGTACACGGCCACCGTACCATGAACGAAGTGACACGTGGAAATATTCAAAAACTCGTGAAAACCGGAATCCAAGAGTCTCTGAAATCGGCGCACCGAAATTTCTCAAGGAGTTCGACATTTTCGATTTCCGAAGAATGCGTGAGATATCTGACGAGATGGTTGTTGGCCGAACGAAGACTTGAACAGCCGTCTGCGGCTATGAATGAATCGTTTGAATTGACCGGTGATAGCAGTAGTAAGAAGAAGGACGATGCCACGTTTGATAGCCTTATCGATCTCTCCTTTGGCTCGACGATTTCCGGAAAACATAAATTGAACGCGTGGAATGGTGTAATGCAAATCCTGAATGAGCTCCTAAAAAGTCGACGACTCGAACTTCAAGTCACTGAAAAAATCGTGACAATCCTCTGGGAAAAGCGAAAATCCTACACAACGGAGCCACTCCGTACTGTGTTCTGCTCCATTCTCTCCACAGTCGTCTGCCAGGCCGATGTTCGATTCGGTCATCGGAAAGTGCCGACAATCGACTCGATTCTCAAATATTCGCTGTCTCTAATGCCAAATGTCGCATCTCTTCCCAGTGCCGCTGCGTTGACCGAAACGATTGTCAGATTCAGGACAGTATCACGAGAGGGTCTCCGTAACACGTGGGATACCGTATCCCGAACTAGCTCCGGCTCATTTGAAGTTGTTCGGCTGATTTCGGCGTTGATCTCCGTTACGGAATTTGATGAGAATTCGAGATTCGCCAACGATGAGAGAGTTCGGAGTTGGAGTTTTCGAAAAGACATAATCGAATGGGTATTGCTGGATCCGAATGCACATAGTCACAAATTACTCTATCAGTTGTGCCAGTATCATCCAACGTATTGTTATGAATCAGAAGCTTCTTCTAGTGACGACTCCCTGCTTCAAACTCTGAAACTATGTAAATTAGCTTGCTCTCCAGCTCCCCCATCGGCTCCAAAAGCCCTCCGACCACTCGAAGCTTCAATTGAAGAGATTGTGAGATATGTGCATGATAAGCTCAAGAGCATTCTCGCGACTGAAATCACTCTGCCTGCATTTGTGCTCTGCCACGAATTTGCTCTGAAGTATCCGGATAGATCTTATGAGTTTAATAAAATGTACAAAAAGCTCTACCAAATCATGGAAGATCAAGAAGAAGACGAGTTTCTCCAATCAGCTCGCCATTTCTCAAAATGGCCTCAAAATCTGACACTACCAATACAAAAACAGACAATAAATTGCATGGCTGTCTTTTTCGAAGCGAATCTTGACAATCAGCTCGTCGATCTCTGTCAGTGGAGTGACCGACGAAAAGTGCTTGTCGAGATGCTCGCCGAGCTGGCCGCCACAAGATCTGAAATTCGAGATAAACTTCAAAAATCGATGCCGTTCAACAAATTCGTCAAGGAGTGTATAATGGAGAATCGCGGTGATTTGTATGAAATGACAAAGAGATTTGAGAAATATTCGTTTTTGCTCTCGATTCGGAATTTAATTGTTACTAGGATGATTATAACAAACGAAGCCGCCCGACTCCTAGGAGATGGTGAAACAATCAGTGAAACCGATATCTTCATAATCGAAAAGCGTACTCTTTCCACGTGTATTCGTAATGTGTCCGAAGGAAAAGAGTTGAGCGGCTACACACTGGACCCCTATACGGTAGCTGCCAACGTGCACAATGTGCATTTCGATCACATAAACGTCGAAATCTATCTGGAATTGCTGAAAAAGTCGCCATTTTTCGCTCAGAACATTGTACGCCACTTGTTACGGCAGAATGGAAAAGAAGCAGAAGAAGAGACGTGGCACTTGCATGCCACTGTGCTGAAAATTGTGATGAAAGATGAAAAGTTGCTGGCGGTATGCGTGGCCACAATTCCAAATATGGTTCGATATCTCAAAGTCTATCAGATTCATTTCAGTCCGAAATCCAACGCGGCGAAGTTCTTACACCTCGACATGGAATCGATTTCCCACTGCCAATCATATTTGCGAAAACCAACAAAATCATCCAATCTGATCACGGCCGCCAACTTTTTGACACTTTTCGGATGTGAAAAGCGCACGTGGAAGCGCCCGATTCTCAGATTTTGGAGCATTTTCAAGCAGCAACCGGCTATGTGTTGCGAGAAATTGCTCATTTTTGCTGAAGAATGTGTCGAACTTGGCCTGAACCACCGAATCGCTTGTCTTTTACGCGCACTGACAACCAGTGAATTCTGCCGAAAAGCTCTATGTGATGAATATCTGAAAATCGCGTTTCAGCTGACTTATCGATCGATTTTTCTGATTTTAAGCAAAAATGAGTGCAGACCAGAAATTGTGGAGCTCTGCGATGACATGAATCTTCGGTACGATCTTCTCCAGCACCAGATCAAACATGTCGCGGCTCACCATTTGGAGCACTTTGAGCGTTTCGAAACGAAAATCGCATTTTCTGTTGAGAAATTTCTGAAATCTGGAATTGATGGAATCGATTTTGAAGATTTAGGATTAGTCGAGTTTTATAAGCAGTTGAACGAAAATTTGACAGAAGACGCGATCAGGAGCAATGAGGCGAGAAACATCTACATCGTCGACATTCTCTCAACAATTTGGCTCCAGCTCCCCTCAATTCGTCCTCAAATTCTACCGATTCTCGCCCGCTTCAAGCACATTTCCCCAGCGTGGACTAATTTCCCACAGCCGCCTCATATTTCGACAAACGAGAAATCATTTCTTCAACATCTACGATTTCATCTTTATCTAAAAATGATGAATATCTCGAAATCCATGACGCAAGGCGAGTATGCCACGTGTATTATGATGCTCCTGACGAGCTACGACTCGAGCCATTTCGTTGCGGATTTGATAGAGAAAAAGCAGCTGGGAAAGCTGAAATTGCAGCAGAGAAGGAATGTTTTGTGCATTTTGAGCCGACTTTTGAAAGATCAAGCTGTGATGGGTGATGAAGATGAGACGATTATCGATCCGATTCTTTTTAAGGCAATCACCAAAGCTTCCGCCGTTTTTGAAGACACCGCCGCGTGCATCGTACCATTTTTGTTCAAAATTTGTGTGGATTTCAAGGGAAAATACGATAAATGTGTGATAAATTTGCTGGGATGTCTTAAGGGAGTTAATGCAGAAGACGAAATAGTAGTCCGCTGCCTTGCCGAATGCGTCGACTCCATTGGACTCAACGTGATTGCTCGTTACGAACGCCTGAACATTGAGACTCATTCGGAATTCGGTGTGAAATGGTTCTTCAAATTATCCCGTCTCTTTCTGAAACATGGATTTACTACGCATTCCTTCGCGATTGCGAATATATTATTTGACCGACTTTCGGCGAGAAAACGAAATACAATGATGATAGATCGTACGAGTTTAGACCGAATTGACAGATCACAGGAACTTATCAATCTTTTGGTGGAGATCTATGTGGCCGAGGGTAACTCGGTAGCGTTGTCCTCTCTGCCACCTGCGGTACAAAATAGACCGGATGTTCGGCAGGTTATGAATAAGAGTTCAAAGGAATGGTTGAAATTGCTGTCTTCGAATCAAATGGACTCGTGGGAGTTGACTATTGTGCAGTGGATGTGTGGCATCCAATTCAATGCAATCACCGGCGATAAATACCTGAATTCAATTCTTCGTTGCAATTTCAACGAGTACACCAAAAAAATCGATTCCCCATTAAAATTCGTCTATTTTCAACTATTCCATCTTTCGACGAGCACTCTGGAAATCGAAGAAGCCATTTCTAGCATGCCGTTGGCTCCAACAATCGATCAAATGCGGCTTATGATTATAGCCAACGCGACGGCAAGCTTCGAGCCACAATCCGTTGAAGAGCACGTTGTTCGAGCTGTTCGAGAGCTCCGAGAGACGTCAAATCGGCGAAAATCCGGTGGAAATGTGAAGGGAATTAATGAAAAAACAACGAGAATGGTGAAACTGGCCGAGATGCTCACCGAGAATAAAGCATACGATGCGGCAATAAACCTGCTCGACACGTGGGAGCACGAGTGTCTCCAATGGACATCTGTCGCTGCCGAATCAATCGATATCGATTTAATTCGAATCTGCAAACAACACGTGACGTGTCGGTCGGGAGATCCAAGAATGGCGGACATAAATCTACGAACAATGCATCCACGTGTCCCGGTGATGAGTGACCTGGCGATTGCCGAATGGTCACTCGCGTTGAGCAAGATTACAATTGAATATCGGAATGATATGGAAGAGGGTATTCGGATTTTGGAATTTGGATGCAAACATTTACAGAATAAGGATTCTGTAGAGACGAGGTTAAAGGTTCTCCTAAAACTCCATTCCGTCTGTATTGGCCAACTGTCGAAACTCGAAGAATATCGCGAAACGCGTACCTACCGTATGAAGCAGCAGGCGGTCACCGCATTCGAACAACAAATTCAAAATTCGTGTCGAACCAGTCTGGCACGTGGCAATTCGGGTGACGAATGGACGAAAAAAACGGTGCAACGGGTGAGAAAAGAGCATCAGTTTGAGAAGAATGATTTGGAAAAAGTGGATAATTCGTTGAATTCGGCGGCCCGGAAAGCTGTATCGTCGGGTTTTGATGCACTTTTATGCATCAGCCAACTGGAAGACGACGACGAAGCGATCCGCGCTTCATCTCTCATAATATTTCCATTAATCGATGTGATCTACAAATACGAAACGGACGTCGGAGTGATCGCCTTGCTCAAAGAGCACACCAAATCGAAGCTCCCGTCGAAGCTGTGGATAAGTGCCACCTCACACATTGCCTCTAAGTGCTTCTCCATCGAAAAATCGCAAATCACGAGACACTTATCACAGATTCTGTGTCATCTCATCTATGACTACCCGTATCACGTTTTGCACACAATTTTGATGTATGATGACGAGAAGAACGCTTCCAAGGTGAAAGGCTTCTTGAAAACGATATTTGACGCGCGAGCTGACCAACGGGATTCGTCGAAGCTTAAGGAGATTGTGATTACTATTCGTGAGGCTCACCAGGCTTATAGAGAAATTGCAATGCTTGACGTGAGAGGAAACGTTCGGATACAACGTGTTGAAATAAACGGAAAAACGATGTACCGATGGCCACATGATTTGAAGATTTTCAAGTGCAAATTGCGTCAATTGCCGATTCCCACCATTTCGCAAAAGATTGGTTGCCCGGGTGACTACTCGACGACTGACCTAATCACGTGGAAACGATGGAAAGATGTATTCACAATTGCCGACGGAATTTCCACTCCAAAAATCTGGGAAATTGAGGGTAGCGATGGAAAATGGTATAAAACTGTGTGGAAGAAGGACGACGTACGACAGGATGTGCTTGTTGAGCAAATGTTTGATGTTACGAATAATATGTTGGAGAAGGCGATGCTTCGGACGTATAATGTTGTCCCGTTAGACACTGAATGTGGAGTTATAGAGTTTTGTGGTGGAACTGTTAGCTTAAAAGAAGTTATGTGTGGTGTGACACGAGAAGGCGGTCTCCACCGGGAGTTCAATTCAGAAGAAGTTTCGGCGAGTAAAGTGTCGTCAATGATGAGACAAGTACAGACAGAGTCCACAGAGACACGACGACAAGTTTTTGTGGAGATTTGTCAGCAGTATTCTCCAGTTTTTAGACACTTCTTCTACACCAACTTTTCGACGGCGCAGATTTGGCGGCAAAAAATCATAAATTACAGGCAGAGCTTGGCCACGTGGAGTATTGTGTGCTACATCGTTGGCCTCGGCGATCGACACGCGTCGAATATTCTATTCGATCAGAAATTGTGCACATTTGTGCATATCGATTTGGGAATGATTTTGGAGTATAGTAAACGAACGTTGCCAGTTCCCGAACAAGTTCCATTCCGTATAACTCGAGATGTGCTCGACCCGATTCTGATTGAAGGAATCGAGAATGGACAGTTGGCTGAGGAATGTACGCAGATTATGGAGAAATTGAAGGAAAATGGAAAGGTAATCCTCGGTGTCGCCTCTGCTCTACTCCGCGAAACGATGACAAATTTCCGAGAAGCCGAACAGGCTGCCGGCCGCCCGTCCTACATTTCTGAAATGGCCATCGGACGGCTTCGAGAAAAGCTACGGGGCACCGATGACGGTGTGACGGCCCAATCGTCGAATCTTCAAATTCGGCGACTTTTACGAGAAGCTACGAGTGCTGACAATTTATCGCGAATGTTCTGCGGATGGATGCCGTTTTTGTAGagaaatcagagaaaatttttctcgaaaattaaaatttttactgtattattgttttctcgtatcaatttttcagaatttgctttgttattttattttacgcgcaaaataatctttctaatttagaaattttaaaattatggaaactttctgaaatttctgcaaaattctagaaaatcaaaaaatttagttttccatcctaattttttttttcgttgattttccccagaaaattcaaacttttactgtattattattttttcatatcatgtaatgttttttgttcaacatttacatgtttttcctacctcttctatgatttccccttcccccaggtctcatttttcttcacaatttttaaaattgattcactgttgaatgtgttgtcttttttctatgcgaattttattaattttatgaaaaaaatttgtaatatttttgtgtaaattttgagtcttcttacactacaaagttaaccatttcagaggagtttcaaaataaatagtgaatttttacaa

Yellow = F56C11.4

Green = K10E9.1

Blue = *atm-1*

Grey = 3’UTR

Revised ATM-1 (*C. elegans*)

MSVTQLKNLKHAIAQLLEWDGTKTARKKIVDEVVLLYHALGAEALSEDNQEIYDLYDLSARIFNLAKKEIEEANQQFEKERKKGTRRSEKPVPTPLFELSIQHLKRCCQQGIDHNQVPWIAYCLKLLEFPITITEKSIENEISNVLLLSSNASQLHWAEHAHLSSLWKWIWSRVETADIGALAMRNYMELAANLLENVDYVVFEKSPIDLMAKVMGTLKKSVEMGNPKEYWCHYRKFSVVHILSYIVHRWGLEARDFILREIFELTGSLSNLISVAHKDGEQSKKCIMRLIDDLVKLAMIETVHGHRTMNEVTRGNIQKLVKTGIQESLKSAHRNFSRSSTFSISEECVRYLTRWLLAERRLEQPSAAMNESFELTGDSSSKKKDDATFDSLIDLSFGSTISGKHKLNAWNGVMQILNELLKSRRLELQVTEKIVTILWEKRKSYTTEPLRTVFCSILSTVVCQADVRFGHRKVPTIDSILKYSLSLMPNVASLPSAAALTETIVRFRTVSREGLRNTWDTVSRTSSGSFEVVRLISALISVTEFDENSRFANDERVRSWSFRKDIIEWVLLDPNAHSHKLLYQLCQYHPTYCYESEASSSDDSLLQTLKLCKLACSPAPPSAPKALRPLEASIEEIVRYVHDKLKSILATEITLPAFVLCHEFALKYPDRSYEFNKMYKKLYQIMEDQEEDEFLQSARHFSKWPQNLTLPIQKQTINCMAVFFEANLDNQLVDLCQWSDRRKVLVEMLAELAATRSEIRDKLQKSMPFNKFVKECIMENRGDLYEMTKRFEKYSFLLSIRNLIVTRMIITNEAARLLGDGETISETDIFIIEKRTLSTCIRNVSEGKELSGYTLDPYTVAANVHNVHFDHINVEIYLELLKKSPFFAQNIVRHLLRQNGKEAEEETWHLHATVLKIVMKDEKLLAVCVATIPNMVRYLKVYQIHFSPKSNAAKFLHLDMESISHCQSYLRKPTKSSNLITAANFLTLFGCEKRTWKRPILRFWSIFKQQPAMCCEKLLIFAEECVELGLNHRIACLLRALTTSEFCRKALCDEYLKIAFQLTYRSIFLILSKNECRPEIVELCDDMNLRYDLLQHQIKHVAAHHLEHFERFETKIAFSVEKFLKSGIDGIDFEDLGLVEFYKQLNENLTEDAIRSNEARNIYIVDILSTIWLQLPSIRPQILPILARFKHISPAWTNFPQPPHISTNEKSFLQHLRFHLYLKMMNISKSMTQGEYATCIMMLLTSYDSSHFVADLIEKKQLGKLKLQQRRNVLCILSRLLKDQAVMGDEDETIIDPILFKAITKASAVFEDTAACIVPFLFKICVDFKGKYDKCVINLLGCLKGVNAEDEIVVRCLAECVDSIGLNVIARYERLNIETHSEFGVKWFFKLSRLFLKHGFTTHSFAIANILFDRLSARKRNTMMIDRTSLDRIDRSQELINLLVEIYVAEGNSVALSSLPPAVQNRPDVRQVMNKSSKEWLKLLSSNQMDSWELTIVQWMCGIQFNAITGDKYLNSILRCNFNEYTKKIDSPLKFVYFQLFHLSTSTLEIEEAISSMPLAPTIDQMRLMIIANATASFEPQSVEEHVVRAVRELRETSNRRKSGGNVKGINEKTTRMVKLAEMLTENKAYDAAINLLDTWEHECLQWTSVAAESIDIDLIRICKQHVTCRSGDPRMADINLRTMHPRVPVMSDLAIAEWSLALSKITIEYRNDMEEGIRILEFGCKHLQNKDSVETRLKVLLKLHSVCIGQLSKLEEYRETRTYRMKQQAVTAFEQQIQNSCRTSLARGNSGDEWTKKTVQRVRKEHQFEKNDLEKVDNSLNSAARKAVSSGFDALLCISQLEDDDEAIRASSLIIFPLIDVIYKYETDVGVIALLKEHTKSKLPSKLWISATSHIASKCFSIEKSQITRHLSQILCHLIYDYPYHVLHTILMYDDEKNASKVKGFLKTIFDARADQRDSSKLKEIVITIREAHQAYREIAMLDVRGNVRIQRVEINGKTMYRWPHDLKIFKCKLRQLPIPTISQKIGCPGDYSTTDLITWKRWKDVFTIADGISTPKIWEIEGSDGKWYKTVWKKDDVRQDVLVEQMFDVTNNMLEKAMLRTYNVVPLDTECGVIEFCGGTVSLKEVMCGVTREGGLHREFNSEEVSASKVSSMMRQVQTESTETRRQVFVEICQQYSPVFRHFFYTNFSTAQIWRQKIINYRQSLATWSIVCYIVGLGDRHASNILFDQKLCTFVHIDLGMILEYSKRTLPVPEQVPFRITRDVLDPILIEGIENGQLAEECTQIMEKLKENGKVILGVASALLRETMTNFREAEQAAGRPSYISEMAIGRLREKLRGTDDGVTAQSSNLQIRRLLREATSADNLSRMFCGWMPFL

***atm-1.a transcript***

ATGAGCGTTACTCAGCTAAAAAATCTGAAACACGCGATAGCTCAGCTTCTGGAGTGGGATGGAACGAAAACGGCGAGAAAGAAAATCGTCGACGAGGTGGTTCTTCTCTATCACGCTCTGGGAGCGGGCATTAAGTGAGGATAACCAGGAGATATATGATTTGTATGATTTATCTGCACGTATATTCAACTTAGCTAAGAAAGAAATCGAAGAAGCCAACCAACAATTCGAAAAAGAACGAAAAAAGGGCACAGCGAAGCGAAAAACCAGTCCCAACTCCACTTTTCGAGCTATCAATACAACATTTAAAACGTTGCTGTCAGCAGGGAATCGATCACAATCAGGTGCCATGGATAGCATATTGTCTGAAACTACTGGATTCCAATCACAATCACCGAAAAATCGATCGAAAACGAGATTTCCAACGTGCTCCTATTGAGCTCCAACGCCTCACAGCTCCATTGGGCCGAGCATGCTCATTTGAGCAGCTTATGGAAATGGATTTGAGCGTGTCGAGACCGCCGATATTGGGGCACTCGCCATGAGAAATTATATGGAATTGGCGGCGAACTTGCTGGAAAACGTGGATTACGTGGTTTTCGAGAAGTCGCCTATTGACCTGATGGCGAAATGATGGAACACTGAAGAAAAGTGTGGAAATGGGAAATCCCAAAGAATATTGGTGTCATTACCGAAAATTCTCGGTCGTCCACATTCTCTCATATATCGTACATCGTTGGGGTCTCGAAGCGAGAGATTCATCTTCGTGAGATTTTCGAGCTCACTGGTAGTTTGTCCAACTTGATATCAGTGGCTCATAAGGATGGAGAACAGTCCAAGAAATGCATAATGCGTTTGATTGATGATCTCGTGAAGCTCGCCAGATCGAACCGTACACGGCCACCGTACCATGAACGAAGTGACACGTGGAAATATTCAAAAACTCGTGAAAACCGGAATCCAAGAGTCTCTGAAATCGGCGCACCGAAATTTCTCAAGGAGTTCGACATTTCGATTCCGAAGAATGCGTGAGATATCTGACGAGATGGTTGTTGGCCGAACGAAGACTTGAACAGCCGTCTGCGGCTATGAATGAATCGTTTGAATTGACCGGTGATAGCAGTAGTAAGAAGAAGACGATGCACGTTTGATAGCCTTATCGATCTCTCCTTTGGCTCGACGATTTCCGGAAAACATAAATTGAACGCGTGGAATGGTGTAATGCAAATCCTGAATGAGCTCCTAAAAAGTCGACGACTCGACTTCAAGTACTGAAAAAATCGTGACAATCCTCTGGGAAAAGCGAAAATCCTACACAACGGAGCCACTCCGTACTGTGTTCTGCTCCATTCTCTCCACAGTCGTCTGCCAGGCCGATGTTCGATTCGTCATCGGAAGTGCCGACAATCGACTCGATTCTCAAATATTCGCTGTCTCTAATGCCAAATGTCGCATCTCTTCCCAGTGCCGCTGCGTTGACCGAAACGATTGTCAGATTCAGGACAGTATCACGGAGGGTCTCCGAACACGTGGGATACCGTATCCCGAACTAGCTCCGGCTCATTTGAAGTTGTTCGGCTGATTTCGGCGTTGATCTCCGTTACGGAATTTGATGAGAATTCGAGATTCGCCAACGATGGAGAGTTCGGAGTGGAGTTTTCGAAAAGACATAATCGAATGGGTATTGCTGGATCCGAATGCACATAGTCACAAATTACTCTATCAGTTGTGCCAGTATCATCCAACGTATTGTTATGAATCAGAACTTCTTCTAGTGAGACTCCCTGCTTCAAACTCTGAAACTATGTAAATTAGCTTGCTCTCCAGCTCCCCCATCGGCTCCAAAAGCCCTCCGACCACTCGAAGCTTCAATTGAAGAGATTGTGAGATAGTGCATGATAAGCTAAGAGCATTCTCGCGACTGAAATCACTCTGCCTGCATTTGTGCTCTGCCACGAATTTGCTCTGAAGTATCCGGATAGATCTTATGAGTTTAATAAAATGTACAAAAAGCTCTCCAAATCATGGAAGACAAGAAGAAGACGAGTTTCTCCAATCAGCTCGCCATTTCTCAAAATGGCCTCAAAATCTGACACTACCAATACAAAAACAGACAATAAATTGCATGGCTGTCTTTTTCGAACGAATCTTGACAATCACTCGTCGATCTCTGTCAGTGGAGTGACCGACGAAAAGTGCTTGTCGAGATGCTCGCCGAGCTGGCCGCCACAAGATCTGAAATTCGAGATAAACTTCAAAAATCGATGCCTTCAACAAATTCGTCAAGAGTGTATAATGGAGAATCGCGGTGATTTGTATGAAATGACAAAGAGATTTGAGAAATATTCGTTTTTGCTCTCGATTCGGAATTTAATTGTTACTAGGATGATTATAAAAACGAAGCCGCCCGACTCTAGGAGATGGTGAAACAATCAGTGAAACCGATATCTTCATAATCGAAAAGCGTACTCTTTCCACGTGTATTCGTAATGTGTCCGAAGGAAAAGAGTTGAGCGGCTACCACTGGACCCCTATACGGTGCTGCCAACGTGCACAATGTGCATTTCGATCACATAAACGTCGAAATCTATCTGGAATTGCTGAAAAAGTCGCCATTTTTCGCTCAGAACATTGTACGCCACTTGTTACGGCAGAATGGAAAAGAAGCAGAAGAAGACGTGGCACTTGCATGCCACTGTGCTGAAAATTGTGATGAAAGATGAAAAGTTGCTGGCGGTATGCGTGGCCACAATTCCAAATATGGTTCGATATCTCAAAGTCTATCAGATTCATTTCAGTCCGAACCAACGCGGCGAAGTTCTTACACCTCGACATGGAATCGATTTCCCACTGCCAATCATATTTGCGAAAACCAACAAAATCATCCAATCTGATCACGGCCGCCAACTTTTTGACACTTTTCGGATGTAAAGCGCACGTGGAAGCGCCCGATTCTCAGATTTTGGAGCATTTTCAAGCAGCAACCGGCTATGTGTTGCGAGAAATTGCTCATTTTTGCTGAAGAATGTGTCGAACTTGGCCTGAACCACCGAATGCTGTCTTTTACGCGCACTGACAACCAGTGAATTCTGCCGAAAAGCTCTATGTGATGAATATCTGAAAATCGCGTTTCAGCTGACTTATCGATCGATTTTTCTGATTTTAAGCAAAAATGAGTGCAACCAAAATTGTGGAGCTCTGCGATGACATGAATCTTCGGTACGATCTTCTCCAGCACCAGATCAAACATGTCGCGGCTCACCATTTGGAGCACTTTGAGCGTTTCGAAACGAAAATCGCATTTTCTTTGAGAATTTCTGAAATCTGGAATTGATGGAATCGATTTTGAAGATTTAGGATTAGTCGAGTTTTATAAGCAGTTGAACGAAAATTTGACAGAAGACGCGATCAGGAGCAATGAGGCGAGAAACATTACATCTCGACATTCTCTCAACAATTTGGCTCCAGCTCCCCTCAATTCGTCCTCAAATTCTACCGATTCTCGCCCGCTTCAAGCACATTTCCCCAGCGTGGACTAATTTCCCACAGCCGCCTCATATTCGACAACGAGAAATCATTTCTTCAACATCTACGATTTCATCTTTATCTAAAAATGATGAATATCTCGAAATCCATGACGCAAGGCGAGTATGCCACGTGTATTATGATGCTCCTGACGAGCTACACTCGAGCATTTCGTTGCGGATTTGATAGAGAAAAAGCAGCTGGGAAAGCTGAAATTGCAGCAGAGAAGGAATGTTTTGTGCATTTTGAGCCGACTTTTGAAAGATCAAGCTGTGATGGGTGATGAGATGAGACGTTATCGATCCGATTCTTTTTAAGGCAATCACCAAAGCTTCCGCCGTTTTTGAAGACACCGCCGCGTGCATCGTACCATTTTTGTTCAAAATTTGTGTGGATTTCAAGGGAAAATACGTAAATGTGTGTAAATTTGCTGGGATGTCTTAAGGGAGTTAATGCAGAAGACGAAATAGTAGTCCGCTGCCTTGCCGAATGCGTCGACTCCATTGGACTCAACGTGATTGCTCGTTACGAACGCCTGACATTGAGACTATTCGGAATTCGGTGTGAAATGGTTCTTCAAATTATCCCGTCTCTTTCTGAAACATGGATTTACTACGCATTCCTTCGCGATTGCGAATATATTATTTGACCGACTTTCGGCGAGAAACGAAATACATGATGATAGATCGTACGAGTTTAGACCGAATTGACAGATCACAGGAACTTATCAATCTTTTGGTGGAGATCTATGTGGCCGAGGGTAACTCGGTAGCGTTGTCCTCTCTGCCACTGCGGTACAAAATGACCGGATGTTCGGCAGGTTATGAATAAGAGTTCAAAGGAATGGTTGAAATTGCTGTCTTCGAATCAAATGGACTCGTGGGAGTTGACTATTGTGCAGTGGATGTGTGGCATCAATTCAATGCAATCCCGGCGATAAATACCTGAATTCAATTCTTCGTTGCAATTTCAACGAGTACACCAAAAAAATCGATTCCCCATTAAAATTCGTCTATTTTCAACTATTCCATCTTTCGACGAGACTCTGGAAATCGAAAAGCCATTTCTAGCATGCCGTTGGCTCCAACAATCGATCAAATGCGGCTTATGATTATAGCCAACGCGACGGCAAGCTTCGAGCCACAATCCGTTGAAGAGCACGTTGTTCAGCTGTTCGAGAGCTCGAGAGACGTCAAATCGGCGAAAATCCGGTGGAAATGTGAAGGGAATTAATGAAAAAACAACGAGAATGGTGAAACTGGCCGAGATGCTCACCGAGAATAAAGCATACGATCGGCAATAAACCTGCTCACACGTGGGAGCACGAGTGTCTCCAATGGACATCTGTCGCTGCCGAATCAATCGATATCGATTTAATTCGAATCTGCAAACAACACGTGACGTGTCGGTCGGGAGATCCAGAATGGCGGACATAAATTACGAACAATGCATCCACGTGTCCCGGTGATGAGTGACCTGGCGATTGCCGAATGGTCACTCGCGTTGAGCAAGATTACAATTGAATATCGGAATGATATGGAAGAGGTATTCGGATTTTGGAATTTGATGCAAACATTTACAGAATAAGGATTCTGTAGAGACGAGGTTAAAGGTTCTCCTAAAACTCCATTCCGTCTGTATTGGCCAACTGTCGAAACTCGAAGAATATCGCAAACGCGTACCTACCGTATGAGCAGCAGGCGGTCACCGCATTCGAACAACAAATTCAAAATTCGTGTCGAACCAGTCTGGCACGTGGCAATTCGGGTGACGAATGGACGAAAAAAACGGTGCAACGGGTGAGAAAAGAGCATCAGTTTGAGAAGAATGATTTGGAAAAAGTGGATAATTCGTTGAATTCGGCGGCCCGGAAAGCTGTATCGTCGGGTTTTGATGCACTTTATTCTGTGTCATCTCATCTAACTACCCGTATCACGTTTTGCACACAATTTTGATGTATGATGACGAGAAGAACGCTTCCAAGGTGAAAGGCTTCTTGAAAACGATATTTGACGCGCGAGCTGACCAACGGGATTCGTCGAAGCTTAGAGATTGTGATTACTATTCGTGAGGCTCACCAGGCTTATAGAGAAATTGCAATGCTTGACGTGAGAGGAAACGTTCGGATACAACGTGTTGAAATAAACGGAAAAACGATGTACCGATGGCCACATATTGAAGATTTTCAAGTGCAAATTGCGTCAATTGCCGATTCCCACCATTTCGCAAAAGATTGGTTGCCCGGGTGACTACTCGACGACTGACCTAATCACGTGGAAACGATGGAAAGATGTATTCACATTCCGACGGAATTTCCACTCCAAAAATCTGGGAAATTGAGGGTAGCGATGGAAAATGGTATAAAACTGTGTGGAAGAAGGACGACGTACGACAGGATGTGCTTGTTGAGCAAATGTTTGATGTTAGAATATATGTTGGAGAAGGCGATGCTTCGGACGTATAATGTTGTCCCGTTAGACACTGAATGTGGAGTTATAGAGTTTTGTGGTGGAACTGTTAGCTTAAAAGAAGTTATGTGTGGTGTGACACGAAAGGCGTCTCCACCGGGAGTTCAATTCAGAAGAAGTTTCGGCGAGTAAAGTGTCGTCAATGATGAGACAAGTACAGACAGAGTCCACAGAGACACGACGACAAGTTTTTGTGGAGATTTGTCAGCATATTCTCAGTTTTTAGACACTTCTTCTACACCAACTTTTCGACGGCGCAGATTTGGCGGCAAAAAATCATAAATTACAGGCAGAGCTTGGCCACGTGGAGTATTGTGTGCTACATCGTTGGCCTCGCGATCGAACGCGTCGAATATTCTATTCGATCAGAAATTGTGCACATTTGTGCATATCGATTTGGGAATGATTTTGGAGTATAGTAAACGAACGTTGCCAGTTCCCGAACAAGTTCCATTCCGTATACTCGAGATTGCTCGACCCGATTCTGATTGAAGGAATCGAGAATGGACAGTTGGCTGAGGAATGTACGCAGATTATGGAGAAATTGAAGGAAAATGGAAAGGTAATCCTCGGTGTCGCCTCTGCTCTCTCCGCGAACGATGACAAATTTCCGAGAAGCCGAACAGGCTGCCGGCCGCCCGTCCTACATTTCTGAAATGGCCATCGGACGGCTTCGAGAAAAGCTACGGGGCACCGATGACGGTGTGACGGCCCATCGTCGAATTTCAAATTCGGCGACTTTTACGAGAAGCTACGAGTGCTGACAATTTATCGCGAATGTTCTGCGGATGGATGCCGTTTTTGTAG

***atm-1.b transcript***

ATGAGCGTTACTCAGCTAAAAAATCTGAAACACGCGATAGCTCAGCTTCTGGAGTGGGATGGAACGAAAACGGCGAGAAAGAAAATCGTCGACGAGGTGGTTCTTCTCTATCACGCTCTGGGAGCGGGCATTAAGTGAGGATAACCAGGAGATATATGATTTGTATGATTTATCTGCACGTATATTCAACTTAGCTAAGAAAGAAATCGAAGAAGCCAACCAACAATTCGAAAAAGAACGAAAAAAGGGCACAGCGAAGCGAAAAACCAGTCCCAACTCCACTTTTCGAGCTATCAATACAACATTTAAAACGTTGCTGTCAGCAGGGAATCGATCACAATCAGGTGCCATGGATAGCATATTGTCTGAAACTACTGGATTCCAATCACAATCACCGAAAAATCGATCGAAAACGAGATTTCCAACGTGCTCCTATTGAGCTCCAACGCCTCACAGCTCCATTGGGCCGAGCATGCTCATTTGAGCAGCTTATGGAAATGGATTTGAGCGTGTCGAGACCGCCGATATTGGGGCACTCGCCATGAGAAATTATATGGAATTGGCGGCGAACTTGCTGGAAAACGTGGATTACGTGGTTTTCGAGAAGTCGCCTATTGACCTGATGGCGAAATGATGGAACACTGAAGAAAAGTGTGGAAATGGGAAATCCCAAAGAATATTGGTGTCATTACCGAAAATTCTCGGTCGTCCACATTCTCTCATATATCGTACATCGTTGGGGTCTCGAAGCGAGAGATTCATCTTCGTGAGATTTTCGAGCTCACTGGTAGTTTGTCCAACTTGATATCAGTGGCTCATAAGGATGGAGAACAGTCCAAGAAATGCATAATGCGTTTGATTGATGATCTCGTGAAGCTCGCCAGATCGAACCGTACACGGCCACCGTACCATGAACGAAGTGACACGTGGAAATATTCAAAAACTCGTGAAAACCGGAATCCAAGAGTCTCTGAAATCGGCGCACCGAAATTTCTCAAGGAGTTCGACATTTCGATTCCGAAGAATGCGTGAGATATCTGACGAGATGGTTGTTGGCCGAACGAAGACTTGAACAGCCGTCTGCGGCTATGAATGAATCGTTTGAATTGACCGGTGATAGCAGTAGTAAGAAGAAGGACGATGCCACGTTTGATAGCCTTATCGATCTCTCCTTTGGCTCGACGATTTCCGGAAAACATAAATTGAACGCGTGGAATGGTGATGCAAATCCTGAATGAGCTCCTAAAAAGTCGACGACTCGAACTTCAAGTCACTGAAAAAATCGTGACAATCCTCTGGGAAAAGCGAAAATCCTACACAACGGAGCCACTCCGTACTGTGTTCTGCCATTCTCTCCACAGTCGTCTGCCAGGCCGATGTTCGATTCGGTCATCGGAAAGTGCCGACAATCGACTCGATTCTCAAATATTCGCTGTCTCTAATGCCAAATGTCGCATCTCTTCCCAGTGCCGCGCTTGACCGAAACGATTGTCAGATTCAGGACAGTATCACGAGAGGGTCTCCGTAACACGTGGGATACCGTATCCCGAACTAGCTCCGGCTCATTTGAAGTTGTTCGGCTGATTTCGGCGTTGATCTCGTACGGAATTTGATGAGAATTCGAGATTCGCCAACGATGAGAGAGTTCGGAGTTGGAGTTTTCGAAAAGACATAATCGAATGGGTATTGCTGGATCCGAATGCACATAGTCACAAATTACTCTATAGTTTGCCAGTATCATCCAACGTATTGTTATGAATCAGAAGCTTCTTCTAGTGACGACTCCCTGCTTCAAACTCTGAAACTATGTAAATTAGCTTGCTCTCCAGCTCCCCCATCGGCTCCAAAAGCCTCCGCCACTCGAAGCTTCAATTGAAGAGATTGTGAGATATGTGCATGATAAGCTCAAGAGCATTCTCGCGACTGAAATCACTCTGCCTGCATTTGTGCTCTGCCACGAATTTGCTCTGAAGTATCGGATAGTCTTATGAGTTTAATAAAATGTACAAAAAGCTCTACCAAATCATGGAAGATCAAGAAGAAGACGAGTTTCTCCAATCAGCTCGCCATTTCTCAAAATGGCCTCAAAATCTGACACTACCATACAAAACAGACAATAAATTGCATGGCTGTCTTTTTCGAAGCGAATCTTGACAATCAGCTCGTCGATCTCTGTCAGTGGAGTGACCGACGAAAAGTGCTTGTCGAGATGCTCGCCGAGCTGGCCGCACAAGATCGAAATTCGAGATAAACTTCAAAAATCGATGCCGTTCAACAAATTCGTCAAGGAGTGTATAATGGAGAATCGCGGTGATTTGTATGAAATGACAAAGAGATTTGAGAAATATTCGTTTTGCTCTCGATCGGAATTTAATTGTTACTAGGATGATTATAACAAACGAAGCCGCCCGACTCCTAGGAGATGGTGAAACAATCAGTGAAACCGATATCTTCATAATCGAAAAGCGTACTCTTTCCACGGTATTCGTAAGTGTCCGAAGGAAAAGAGTTGAGCGGCTACACACTGGACCCCTATACGGTAGCTGCCAACGTGCACAATGTGCATTTCGATCACATAAACGTCGAAATCTATCTGGAATTGCTGAAAAGTCGCCATTTTCGCTCAGAACATTGTACGCCACTTGTTACGGCAGAATGGAAAAGAAGCAGAAGAAGAGACGTGGCACTTGCATGCCACTGTGCTGAAAATTGTGATGAAAGATGAAAAGTTGCGGCGGTATGCGTGCCACAATTCCAAATATGGTTCGATATCTCAAAGTCTATCAGATTCATTTCAGTCCGAAATCCAACGCGGCGAAGTTCTTACACCTCGACATGGAATCGATTTCCCACTGCCAACATATTTGCGAAACCAACAAAATCATCCAATCTGATCACGGCCGCCAACTTTTTGACACTTTTCGGATGTGAAAAGCGCACGTGGAAGCGCCCGATTCTCAGATTTTGGAGCATTTTCAAGCAGCACCGGCTATGTGTTGGAGAAATTGCTCATTTTTGCTGAAGAATGTGTCGAACTTGGCCTGAACCACCGAATCGCTTGTCTTTTACGCGCACTGACAACCAGTGAATTCTGCCGAAAAGCTCTATGTGTGAATATCTGAAAATGCGTTTCAGCTGACTTATCGATCGATTTTTCTGATTTTAAGCAAAAATGAGTGCAGACCAGAAATTGTGGAGCTCTGCGATGACATGAATCTTCGGTACGATCTTCTCCAGACCAGATCAAACATGTGCGGCTCACCATTTGGAGCACTTTGAGCGTTTCGAAACGAAAATCGCATTTTCTGTTGAGAAATTTCTGAAATCTGGAATTGATGGAATCGATTTTGAAGATTTAGGATTGTCGAGTTTTATAAGCATTGAACGAAAATTTGACAGAAGACGCGATCAGGAGCAATGAGGCGAGAAACATCTACATCGTCGACATTCTCTCAACAATTTGGCTCCAGCTCCCCTCAATTCGTCCTCAATTCTACCGATTCTCGCCGCTTCAAGCACATTTCCCCAGCGTGGACTAATTTCCCACAGCCGCCTCATATTTCGACAAACGAGAAATCATTTCTTCAACATCTACGATTTCATCTTTATCTAAAATGATGAATATCTCGAAATCATGACGCAAGGCGAGTATGCCACGTGTATTATGATGCTCCTGACGAGCTACGACTCGAGCCATTTCGTTGCGGATTTGATAGAGAAAAAGCAGCTGGGAAAGCTGAATTGCAGCAGAGAAGGAATGTTTGTGCATTTTGAGCCGACTTTTGAAAGATCAAGCTGTGATGGGTGATGAAGATGAGACGATTATCGATCCGATTCTTTTTAAGGCAATCACCAAAGCTTCCGCCGTTTTTGAAGACACCGCCGCGTGCATCGTACCATTTTTGTTCAAAATTTGTGTGGATTTCAAGGGAAAATACGATAAATGTGTGATAAATTTGCTGGGATGTCTTAAGGGAGTTAATGCAGAAGAAAATAGTAGTCCGCTGCCTTGCCGAATGCGTCGACTCCATTGGACTCAACGTGATTGCTCGTTACGAACGCCTGAACATTGAGACTCATTCGGAATTCGGTGTGAAATGGTTCTTCAAATTATCCCTTCTTTCTGAAACATGGATTTACTACGCATTCCTTCGCGATTGCGAATATATTATTTGACCGACTTTCGGCGAGAAAACGAAATACAATGATGATAGATCGTACGAGTTTAGACCGAATTGACAGACAAGGAACTTATCAATCTTTTGGTGGAGATCTATGTGGCCGAGGGTAACTCGGTAGCGTTGTCCTCTCTGCCACCTGCGGTACAAAATAGACCGGATGTTCGGCAGGTTATGAATAAGAGTTCAAAGAAGGTTGAAATTGCTGTCTTCGAATCAAATGGACTCGTGGGAGTTGACTATTGTGCAGTGGATGTGTGGCATCCAATTCAATGCAATCACCGGCGATAAATACCTGAATTCAATTCTTCGTTGCATTTCACGAGTACACCAAAAAAATCGATTCCCCATTAAAATTCGTCTATTTTCAACTATTCCATCTTTCGACGAGCACTCTGGAAATCGAAGAAGCCATTTCTAGCATGCCGTTGGCTCCAACAATCATCAATGCGGCTTATGATTATAGCCAACGCGACGGCAAGCTTCGAGCCACAATCCGTTGAAGAGCACGTTGTTCGAGCTGTTCGAGAGCTCCGAGAGACGTCAAATCGGCGAAAATCCGGTGGAAAGTGAAGGAATTAATGAAAAAACAACGAGAATGGTGAAACTGGCCGAGATGCTCACCGAGAATAAAGCATACGATGCGGCAATAAACCTGCTCGACACGTGGGAGCACGAGTGTCTCCAATGGACATTGTCGCTCCGAATCAATCGATATCGATTTAATTCGAATCTGCAAACAACACGTGACGTGTCGGTCGGGAGATCCAAGAATGGCGGACATAAATCTACGAACAATGCATCCACGTGTCCCGGTGATGGTGACCTGCGATTGCCGAATGGTCACTCGCGTTGAGCAAGATTACAATTGAATATCGGAATGATATGGAAGAGGGTATTCGGATTTTGGAATTTGGATGCAAACATTTACAGAATAAGGATTCTGTGAGACGAGGTAAAGGTTCTCCTAAAACTCCATTCCGTCTGTATTGGCCAACTGTCGAAACTCGAAGAATATCGCGAAACGCGTACCTACCGTATGAAGCAGCAGGCGGTCACCGCATTCGAACAACAATTCAAAATCGTGTCGAACCAGTCTGGCACGTGGCAATTCGGGTGACGAATGGACGAAAAAAACGGTGCAACGGATTCTGTGTCATCTCATCTATGACTACCCGTATCACGTTTTGCACACAATTTGATGTATGATACGAGAAGAACGCTTCCAAGGTGAAAGGCTTCTTGAAAACGATATTTGACGCGCGAGCTGACCAACGGGATTCGTCGAAGCTTAAGGAGATTGTGATTACTATTCGTGAGGCTCACAGGCTTATAGAAAATTGCAATGCTTGACGTGAGAGGAAACGTTCGGATACAACGTGTTGAAATAAACGGAAAAACGATGTACCGATGGCCACATGATTTGAAGATTTTCAAGTGCAAATTGCGTCATTGCCGATTCCCCCATTTCGCAAAAGATTGGTTGCCCGGGTGACTACTCGACGACTGACCTAATCACGTGGAAACGATGGAAAGATGTATTCACAATTGCCGACGGAATTTCCACTCCAAAAATCGGGAAATTGAGGGTGCGATGGAAAATGGTATAAAACTGTGTGGAAGAAGGACGACGTACGACAGGATGTGCTTGTTGAGCAAATGTTTGATGTTACGAATAATATGTTGGAGAAGGCGATGCTTCGACGTATAATGTTGTCCGTTAGACACTGAATGTGGAGTTATAGAGTTTTGTGGTGGAACTGTTAGCTTAAAAGAAGTTATGTGTGGTGTGACACGAGAAGGCGGTCTCCACCGGGAGTTCAATTCAGAGAAGTTTCGGCGAGTAAGTGTCGTCAATGATGAGACAAGTACAGACAGAGTCCACAGAGACACGACGACAAGTTTTTGTGGAGATTTGTCAGCAGTATTCTCCAGTTTTTAGACACTTCTTCTACCCAACTTTTCGACGGCGAGATTTGGCGGCAAAAAATCATAAATTACAGGCAGAGCTTGGCCACGTGGAGTATTGTGTGCTACATCGTTGGCCTCGGCGATCGACACGCGTCGAATATTCTATTCGACAGAAATTGTGCACATTTTGCATATCGATTTGGGAATGATTTTGGAGTATAGTAAACGAACGTTGCCAGTTCCCGAACAAGTTCCATTCCGTATAACTCGAGATGTGCTCGACCCGATTCTGATTGAGGAATCGAGAATGGACAGTGGCTGAGGAATGTACGCAGATTATGGAGAAATTGAAGGAAAATGGAAAGGTAATCCTCGGTGTCGCCTCTGCTCTACTCCGCGAAACGATGACAAATTTCCGAGAACCGAACAGGCTGCCGGCCGCCGTCCTACATTTCTGAAATGGCCATCGGACGGCTTCGAGAAAAGCTACGGGGCACCGATGACGGTGTGACGGCCCAATCGTCGAATCTTCAAATTCGGCGACTTTTACGAGAAGCTACGAGTGCTGACAATTTATCGCGAATGTTCTGCGGATGGACCGTTTTTGTAG
